# Intravitreal Anti-vascular Endothelial Growth Factor (Anti-VEGF) Therapy for Valsalva Related Sub-internal Limiting Membrane (Sub-ILM) Macular Haemorrhage: A Case Report

**DOI:** 10.7759/cureus.44420

**Published:** 2023-08-30

**Authors:** Suhaifi Nur-Najwa, Embong Zunaina, Yaakub Azhany

**Affiliations:** 1 Department of Ophthalmology and Visual Science, School of Medical Sciences, Universiti Sains Malaysia, Kubang Kerian, MYS; 2 Eye Clinic, Hospital Universiti Sains Malaysia, Kubang Kerian, MYS

**Keywords:** ranibizumab, anti-vegf, premacular haemorrhage, sub-ilm haemorrhage, valsalva retinopathy

## Abstract

Valsalva retinopathy is an uncommon type of retinopathy that manifests as a rapid and painless vision decline, typically observed in young individuals without prior medical conditions. This condition arises from an elevated pressure within the veins of the eye, causing preretinal haemorrhage with a notable tendency to impact the macula. We describe here a case of valsalva-related sub-internal limiting membrane (sub-ILM) macular haemorrhage which was successfully treated with anti-vascular endothelial growth factor (anti-VEGF). A 27-year-old woman presented with a clinical presentation of a large sub-ILM macular haemorrhage resulting from a valsalva maneuver following a prolonged severe cough. The sub-ILM macular haemorrhage was completely resolved after being treated with three injections of intravitreal ranibizumab with a visual recovery of vision from counting fingers to 20/20 on three month follow-up.

## Introduction

Valsalva retinopathy is a type of retinopathy distinguished by the presence of preretinal hemorrhage. It was initially described by Duane et al. in 1972 [1]. The occurrence of preretinal hemorrhage is a consequence of the abrupt elevation in intraocular venous pressure, resulting in the rupture of perifoveal superficial retinal capillaries. This phenomenon is triggered by an upsurge in intrathoracic or intraabdominal pressure, typically occurring during a Valsalva maneuver [1].

In cases of Valsalva retinopathy, the blood often accumulates in the potential space under the internal limiting membrane (ILM); sub-ILM haemorrhage, or between the ILM and hyaloid face; subhyaloid haemorrhage at or around the fovea [2]. The main approaches to treatment encompass the following methods: observation, intravitreal administration of antiangiogenic medications, neodymium-doped yttrium aluminum garnet (Nd:YAG) hyaloidotomy, pars plana vitrectomy, and intravitreal injection of gas with or without tissue plasminogen activator [2,3]. In this case report, we describe the successful use of intravitreal anti-vascular endothelial growth factor (VEGF) in the treatment of sub-ILM macular haemorrhage due to Valsalva retinopathy.

## Case presentation

A 27-year-old woman noticed a sudden decreased central vision in her left eye upon waking up from sleep, and she presented later on the same day of the onset of decreased vision. She had been experiencing a mild but persistent productive cough, which was particularly pronounced at night. At times, the cough reached a level of severity that induced abdominal pain. This coughing persisted even during sleep until the day of her clinic visit. There were no identifiable factors that could have triggered her coughing. There was no account of any preceding trauma or engagement in heavy lifting activities before the onset of her reduced vision. She has no history of constipation or vomiting. Her previous medical history was unremarkable with no history of bleeding tendency. She is a non-smoker and non-asthmatic. She received antibiotic treatment from a private general practitioner; however, it took several weeks for her symptoms to resolve.

**Figure 1:**
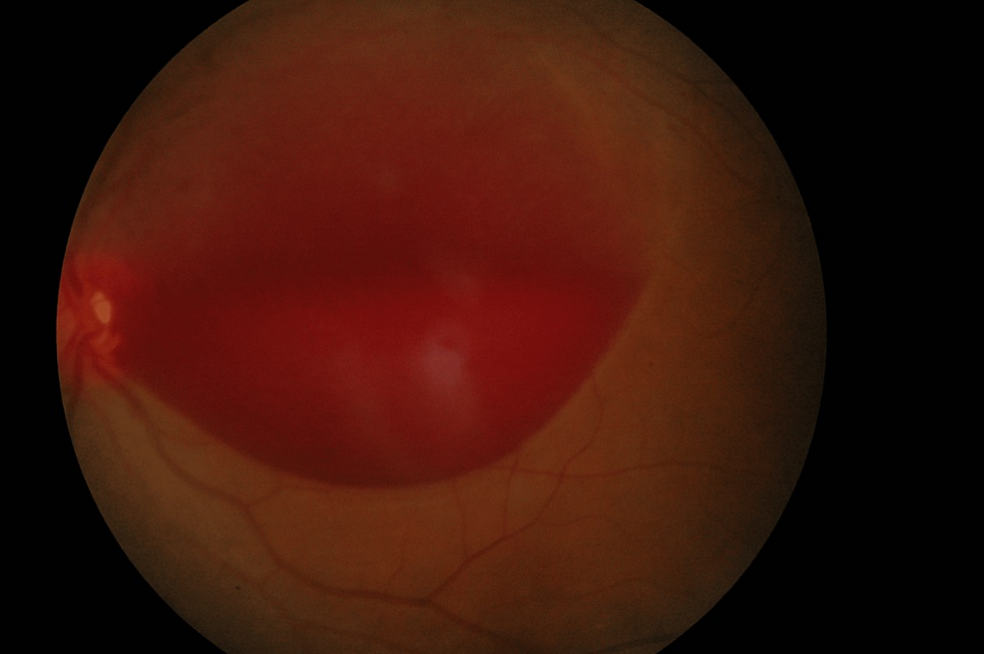
The left eye fundus image The left eye fundus examination revealed a dome-shaped sub-internal limiting membrane haemorrhage at the macula during the initial presentation.

The ophthalmic assessment revealed that the right eye had a best corrected visual acuity of 20/20, while the left eye could only perceive hand movements at a distance of two meters. The pupillary reaction was normal, and there was no evidence of a relative afferent pupillary defect. The anterior segment evaluations were normal. The intraocular pressure (IOP) was 14 mm Hg bilaterally with a Goldmann tonometer. The left fundus examination revealed a round dark red preretinal haemorrhage with a convex surface covering the left macula, which was approximately 12-to-14-disc diameter (Figure 1). The premacular haemorrhage was dense inferiorly, with more diffuse blood superiorly. There was no posterior vitreous detachment detected with a dilated eye examination. The retinal vessels were normal with no new vessel or vasculitis changes. There was no vitreous haemorrhage, submacular haemorrhage, or retinal/subretinal exudate. The right fundus examination was normal.

General examination showed the absence of petechiae or ecchymoses. There was no lymphadenopathy with no hepatomegaly or splenomegaly. Throat examination revealed no injected or enlarged tonsils. Respiratory system revealed normal breath sounds with no rhonchi or crepitation. The cardiovascular system was normal. 

**Figure 2:**
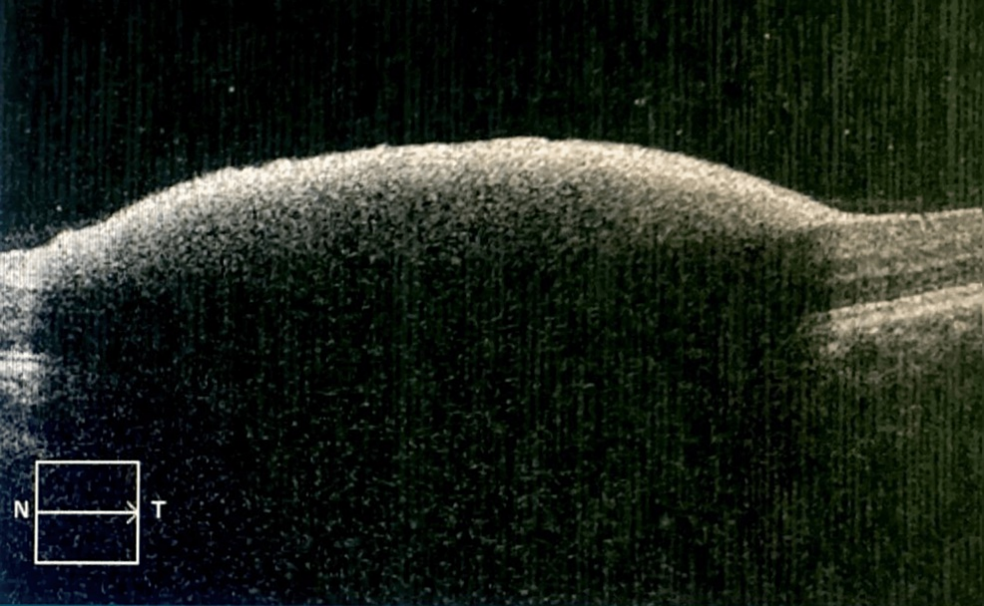
The initial optical coherence tomography scan image of the left eye The initial optical coherence tomography scan revealed a dome-shaped haemorrhage with an area of hyperreflectivity under the internal limiting membrane at the macular area.

Optical coherence tomography (OCT) scan of the left eye through the haemorrhage revealed a dome-shaped haemorrhage with an area of hyperreflectivity under the ILM (Figure 2). The right eye scan was normal.

Blood pressure and baseline haematological investigations such as random blood sugar, complete blood count, and coagulation profile were within normal range. The neutrophil, lymphocyte, and eosinophil differential counts were within normal range. Tests for tuberculosis were conducted, including sputum acid-fast bacilli (AFB) and chest X-ray, all of which yielded normal results. Her COVID-19 test also came back negative. Based on the clinical findings and laboratory studies, the patient was diagnosed to have Valsalva retinopathy of the left eye.

Due to the extensive sub-ILM hemorrhage involving the macular region, the patient was recommended to undergo Nd:YAG laser hyaloidotomy as the initial therapeutic approach. However, she declined this option. Nonetheless, the patient consented to the administration of intravitreal ranibizumab injections to expedite the absorption of the blood, thereby accelerating visual recuperation and reducing potential scarring impact on the macula.

**Figure 3:**
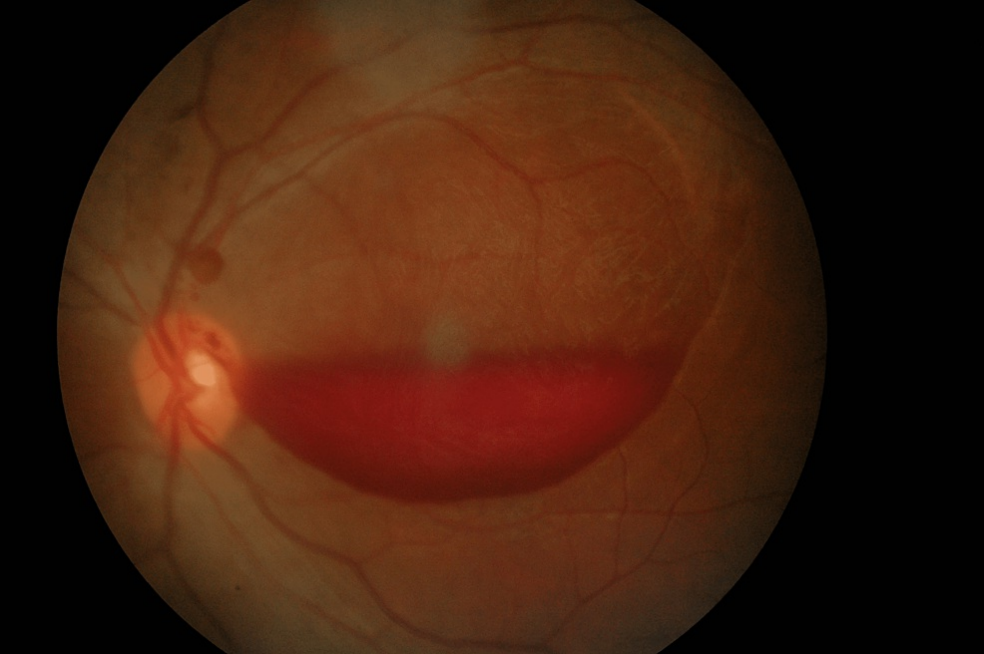
At four weeks following the first intravitreal ranibizumab injection of the left eye A partial resolution in the sub-internal limiting membrane macular hemorrhage was observed four weeks after the first intravitreal ranibizumab injection.

There was a significant partial resolution of sub-ILM macular haemorrhage at four weeks following the first intravitreal ranibizumab 0.5mg (0.05ml) injection (Figure 3), which is at six weeks after initial presentation with the improvement of vision from counting finger to 20/400.

**Figure 4:**
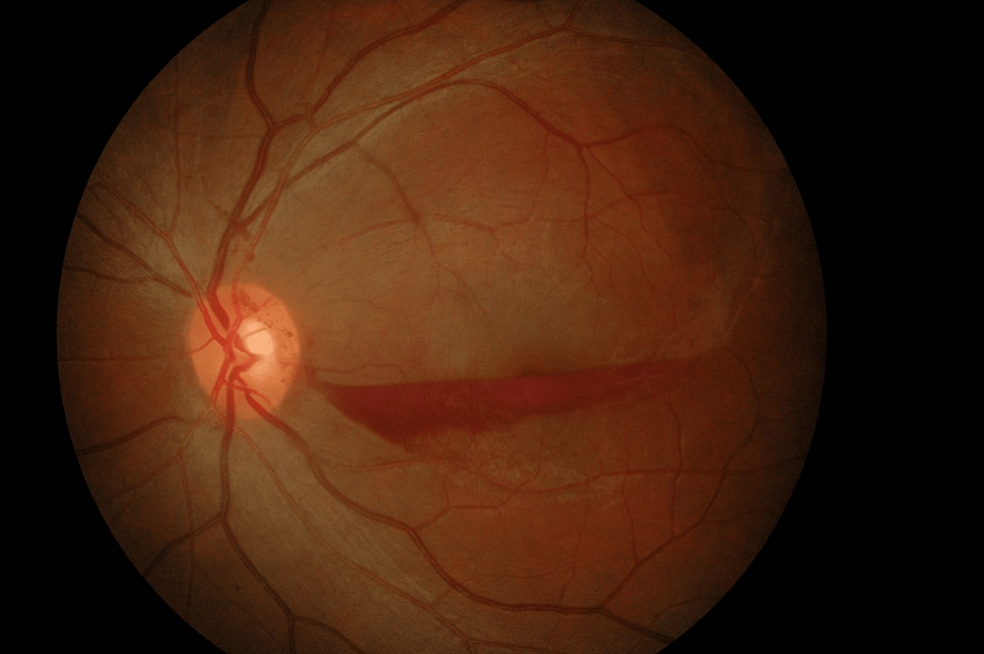
At two weeks following the second intravitreal ranibizumab injection of the left eye, The minimal residual of sub-internal limiting membrane macular haemorrhage was observed after two weeks following the second intravitreal ranibizumab injection.

The left eye visual acuity further improved to 20/50 with minimal residual sub-ILM macular haemorrhage just two weeks after the second intravitreal ranibizumab injection (Figure 4), which is at 10 weeks after the initial presentation.

**Figure 5:**
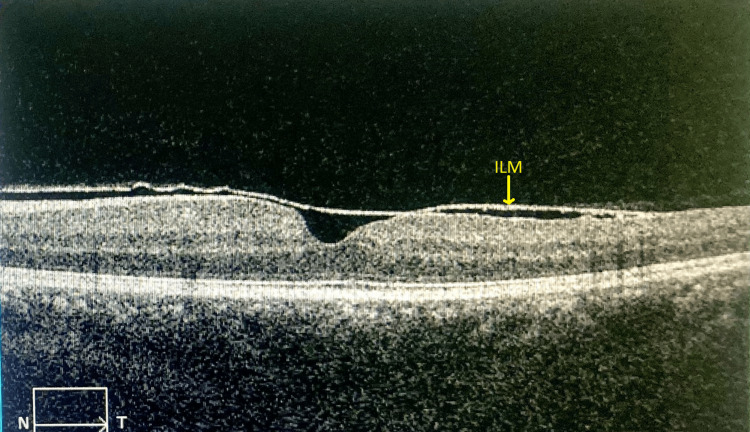
Optical coherence tomography scan of the left eye at three months Optical coherence tomography scan revealed resolved sub-internal limiting membrane macular haemorrhage with a detached internal limiting membrane from retinal layers at the left fovea and parafovea area.

At the three-month follow-up; four weeks after the third intravitreal ranibizumab, the left eye visual acuity improved to 20/20 with complete resolution of sub-ILM macular haemorrhage. She did not complain of metamorphopsia. The OCT showed resolved sub-ILM haemorrhage with detached ILM from retinal layers at the fovea and parafovea areas (Figure 5).

## Discussion

Valsalva retinopathy can result in sudden, severe central vision loss due to the premacular location of the resulting sub-ILM haemorrhage. Vascular diseases of the retina; diabetic retinopathy (30%), Valsalva haemorrhagic retinopathy (30%), and thrombosis of the central retinal vein (20%) are the most common causes of subhyaloid or sub-ILM haemorrhage [4]. Premacular haemorrhages less commonly occur in hematological diseases, eye trauma, macroaneurysm rupture, and Terson's syndrome [5].

Valsalva retinopathy is described as an increase in intrathoracic pressure against the closed glottis, resulting in an increase in cranial venous pressure above the neck. The absence of venous valves in the neck leads to a sudden rise in intraocular venous pressure, subsequently causing the rupture of perifoveal superficial retinal capillaries [6]. This results in haemorrhagic detachment of the ILM or subhyaloid space, leading to a sudden loss of vision, particularly if located in the macular region [7].

Various treatment approaches can be used to manage a sub-ILM haemorrhage, such as observation for spontaneous absorption, intravitreal injection of recombinant tissue plasminogen activator (rt-PA) with gas, pars plana vitrectomy, or laser application (argon or Nd:YAG laser) [5,7,8]. The use of Intravitreal injection of anti-VEGF (ranibizumab) is not well established. VEGF inhibitors with anti-inflammatory and anti-angiogenesis properties are believed to promote the clearance of sub-ILM haemorrhage.

Clinical observation for the spontaneous resorption of sub-ILM haemorrhage is possible and may take several weeks up to six months, depending on severity [8]. However, the risk of the formation of proliferative vitreoretinopathy, epiretinal membrane, and preretinal tractional membrane due to the prolonged existence of haemorrhage and the toxicity of blood breakdown products to the retina may cause incomplete restoration of visual functions [7,9].

Nd:YAG laser hyaloidectomy is a minimally invasive treatment and is useful for non-coagulated and non-dense macular haemorrhages. It leads to a rapid improvement in vision by clearing the obstructed macular area, allowing the hemorrhage to drain into the vitreous cavity, and promoting the reabsorption of blood cells. However, potential complications such as the development of macular holes, epiretinal membranes, or retinal detachment may occur [7,10]. Nonetheless, using a YAG laser for drainage becomes unfeasible when the premacular blood has undergone coagulation [11]. 

Ranibizumab is a recombinant humanized immunoglobulin monoclonal antibody fragment with a molecular weight of 48 kD. It functions by attaching to human VEGF-A and preventing interaction with its receptor. Upon intravitreal administration, the fragment efficiently traverses through all retinal layers, leading to a reduction in cell proliferation, vascular permeability, and the development of choroidal neovascularization (CNV) [12]. It has been used in the treatment of premacular haemorrhage in cases of wet age-related macular degeneration, diabetic retinopathy, myopic CNV, and retinal vein occlusion [13]. Noorlaila et al. [14] reported using anti-VEGF therapy for premacular haemorrhage secondary to Valsalva retinopathy. In their case report, the patient had dramatic visual recovery at one month after intravitreal ranibizumab [14].

In our presented case, a young woman exhibited a substantial and extensive sub-ILM hemorrhage in the macular region, which could have a prolonged clearance period and significantly impede the patient's quality of life. Hence, treatment consideration becomes crucial in this scenario. Notably, the sub-ILM hemorrhage showed nearly complete resolution within 10 weeks following two intravitreal ranibizumab injections. As a result, her vision improved to 20/20 during the three-month follow-up after the initial presentation. Although spontaneous hemorrhage resolution is feasible, the absorption may extend over several months; primarily due to the extensive nature of the haemorrhage. Hence, the utilization of anti-VEGF injections expedited the process of resolving the sub-ILM macular hemorrhage in our patient.

The mechanism of this therapeutic effect in Valsalva retinopathy is not well understood but can be hypothesized as VEGF inhibitors with anti-inflammatory and anti-angiogenesis properties which promote the clearance of sub-ILM haemorrhage. Persistent subhyaloid bleed leads to damage of photoreceptors mediated by iron toxicity, impairment of diffusion of oxygen, and mechanical damage due to clot contraction [9]. Further assessment is required to better understand the effectiveness of anti-VEGF treatment for premacular hemorrhage.

## Conclusions

The use of intravitreal anti-VEGF injections in cases of Valsalva retinopathy as an alternative treatment would be based on the severity of the hemorrhage, the presence of any associated macular edema, and the potential impact on visual function. If there is significant macular involvement or prolonged bleeding, intravitreal anti-VEGF therapy might be considered to help reduce inflammation, promote healing, and resolution of the haemorrhage.
